# Distinct Types of Regulated Cell Death in Melanoma

**DOI:** 10.3390/cells14110823

**Published:** 2025-06-01

**Authors:** Qi Wu, Shuang Liang, Guo-Jun Shi, Guo-Liang Meng, Sheng-Ju Yang

**Affiliations:** 1 Department of Pharmacology, School of Pharmacy, Nantong University, Nantong 226001, China; 2231320314@stmail.ntu.edu.cn (Q.W.); 2119331244@stmail.ntu.edu.cn (G.-J.S.); 2 Medical School, Nantong University, Nantong 226001, China; 2331320318@stmail.ntu.edu.cn

**Keywords:** melanoma, regulatory cell death, apoptosis, autophagy, pyroptosis, immunogenic cell death, necroptosis, ferroptosis

## Abstract

Resistance to cell death is one of the core hallmarks of cancer, with regulatory abnormalities particularly pronounced in the malignant progression and therapeutic resistance of melanoma. This review aims to systematically summarize the roles and mechanisms of regulated cell death (RCD) in melanoma. Currently, distinct types of RCD, including apoptosis, autophagy, pyroptosis, immunogenic cell death, necroptosis, and ferroptosis, have all been found to be involved in melanoma. Autophagy promotes the survival of melanoma cells under stress conditions through metabolic adaptation, yet its excessive activation can trigger cell death. Immunogenic cell death has the capacity to elicit adaptive immune responses in immunocompetent syngeneic hosts. Necroptosis, governed by the receptor-interacting protein kinase 1 (RIPK1)/RIPK3 mixed lineage kinase domain-like protein (MLKL) signaling axis, can synergize with immunotherapy to enhance anti-melanoma immune responses when activated. Pyroptosis, mediated by Gasdermin proteins, induces the release of inflammatory factors that reshape the tumor microenvironment and enhance the efficacy of immune checkpoint inhibitors. Ferroptosis, characterized by lipid peroxidation, can overcome melanoma resistance by targeting the solute carrier family 7 member 11 (SLC7A11)/glutathione peroxidase 4 (GPX4) axis. Therapeutic strategies targeting RCD pathways have demonstrated breakthrough potential. Several agents have been developed to target RCD in order to suppress melanoma.

## 1. Introduction

Melanoma is a malignant tumor originating from cutaneous or non-cutaneous melanocytes. It is characterized by its high invasiveness, malignancy, and poor prognosis, with a rising incidence observed globally [1,2]. The Global Cancer Observatory (GLOBOCAN) reports that melanoma ranks 17th in incidence and 22nd in mortality among all malignant tumors [3,4]. Currently, treatment options for melanoma have expanded beyond surgical resection to include small-molecule targeted therapies and immunotherapies, which have contributed to improving the overall patient prognosis. However, the survival rate for patients with advanced melanoma who develop drug resistance due to prolonged treatment remains alarmingly low [5,6,7,8]. It is important to recognize that both intrinsic resistance, resulting from clonal evolution, and acquired resistance, which arises from the activation of alternative survival pathways, are complex processes influenced by tumor subtypes, genotypes, heterogeneity, and individual patient-specific characteristics [9,10].

Since its establishment in 2005, the Nomenclature Committee on Cell Death (NCCD) has systematically defined and classified cell death from various perspectives [11]. Functionally, cell death is divided into two categories: accidental cell death (ACD) and regulated cell death (RCD). ACD refers to an uncontrolled process of cell death that occurs due to severe physical, chemical, or mechanical damage. Conversely, RCD is a regulated process that arises from imbalances in cellular adaptive responses, which are triggered by excessive or prolonged disturbances in the intracellular or extracellular microenvironments. This process involves multiple signaling molecules and their associated signaling cascades [11,12,13]. Currently, various forms of RCD including apoptosis, autophagy, pyroptosis, immunogenic cell death, necroptosis, and ferroptosis have been implicated in the pathology of melanoma [14]. Understanding the relationships between the relevant targets and signaling pathways associated with these modes of cell death, as well as their roles in the pathogenesis and progression of melanoma, may yield novel insights for therapeutic interventions. Moreover, there were overlapping mechanisms between different types of cell death [15,16]. Previous reviews have predominantly concentrated on the role of individual mechanisms of cell death in melanoma. Our present review summarizes the distinct coexisting types of RCD during the development of melanoma, elucidates their interactions, and compiles therapeutic strategies targeting these pathways for the treatment of melanoma.

## 2. Apoptosis in Melanoma

Apoptosis refers to a regulated process of cell death that is triggered by various stimuli, by extrinsic or intrinsic pathways. Apoptosis is essential for the preservation of normal physiological functions and the maintenance of tissue homeostasis [17]. The primary morphological alterations associated with apoptosis include cellular shrinkage, chromatin condensation, membrane blebbing, nuclear fragmentation, and the formation of apoptotic bodies (ABs), all of which ultimately contribute to the death of melanoma cells [18].

The extrinsic apoptotic pathway is primarily facilitated by two categories of receptors located on the cell membrane. The first category consists of death receptors (DRs), which engage Fas-associated protein with death domain (FADD) and subsequently associate with pro-Caspase-8 to establish a death-inducing signaling complex (DISC), ultimately leading to the activation of Caspase-8. The second category comprises pattern recognition receptors (PRRs), which interact with pathogen-associated molecular patterns (PAMPs) to activate Caspases-9. The intrinsic apoptotic pathway is primarily regulated by mitochondrial processes, and is triggered by a range of stimuli, including DNA damage, hypoxia, oxidative stress, intracellular acidosis, and endoplasmic reticulum (ER) stress [19]. These stimuli lead to the enhanced p53 level and the upregulation of pro-apoptotic proteins such as BH3-interacting domain death agonist (Bid), B-cell lymphoma-2 (Bcl-2) modifying factor (BMF), p53 upregulated modulator of apoptosis (PUMA), Bcl-2-interacting mediator of cell death (BIM), and Bcl-2 associated agonist of cell death (BAD) [20]. These pro-apoptotic proteins interact with anti-apoptotic proteins, including Bcl-2 and BFL-1, facilitating the release of Bcl-2 antagonist/killer (BAK) and Bcl-2-associated X protein (BAX). This interaction promotes mitochondrial outer membrane permeabilization (MOMP) and the subsequent release of various pro-apoptotic factors, such as cytochrome c (Cyto-C), second mitochondrial activator of caspases (SMAC), HTRA serine peptidase 2 (HTRA2), apoptosis-inducing factor (AIF), and endonuclease G (EndoG). Specifically, Cyto-C binds to apoptotic peptidase activating factor 1 (APAF1) and pro-Caspase-9, resulting in the formation of the apoptosome, which activates Caspase-9 and the executioner caspases (Caspases-3/6/7), thereby initiating the apoptotic cascade. Concurrently, SMAC enhances apoptosis by inhibiting the X-linked inhibitor of apoptosis (XIAP), an anti-apoptotic protein that can diminish the activity of executioner caspases. It is noteworthy that these executioner caspases promote apoptosis by activating DNase and Rho-associated protein kinase 1 (ROCK-1), which are responsible for chromatin condensation and DNA fragmentation, as well as membrane blebbing, respectively. Following these processes, apoptotic cells are ultimately fragmented into smaller structures known as ABs. Furthermore, AIF and EndoG can induce apoptosis independently of caspases by translocating to the nucleus, where they cleave DNA, thereby contributing to the apoptotic process [21,22,23,24]. It is important to note that the inhibition of apoptosis, resistance to apoptotic signals, and evasion of immune surveillance during the apoptotic process often contribute to tumor development and progression.

**Figure 1:**
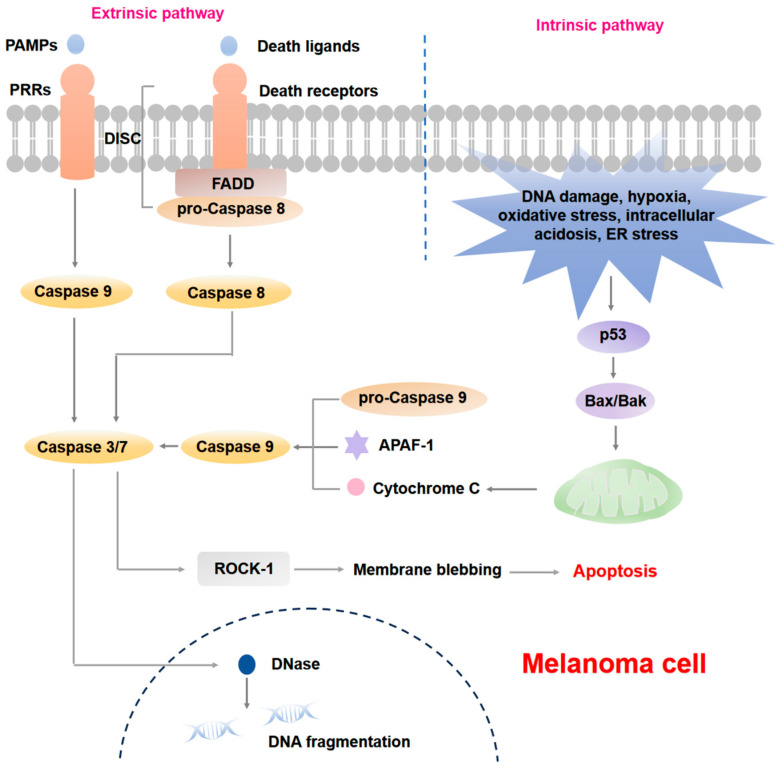
Potential molecular mechanisms of apoptosis in melanoma. The extrinsic apoptotic pathway is initiated by two types of receptors on the cell membrane: death receptors (DRs) which engage Fas-associated protein with death domain (FADD) and associate with pro-Caspase-8 to form a death-inducing signaling complex (DISC), ultimately leading to the activation of Caspase-8, and pattern recognition receptors (PRRs) which respond to pathogen-associated molecular patterns (PAMPs) to activate Caspases-9. The intrinsic apoptotic pathway is primarily regulated by mitochondrial processes. In response to various stimuli such as DNA damage, hypoxia, oxidative stress, intracellular acidosis and endoplasmic reticulum (ER) stress, the levels of p53 protein rise substantially, which will activate Bcl-2 antagonist/killer (BAK) and Bcl-2-associated X protein (BAX), leading to the release of cytochrome c (Cyto-C) from the mitochondria, which then forms an apoptosome with apoptotic peptidase activating factor 1 (APAF1) and pro-Caspase-9, which activates Caspase-9 and executioner caspases (Caspases-3/7). These executioner caspases facilitate apoptosis by activating DNase and Rho-associated protein kinase 1 (ROCK-1), which are responsible for chromatin condensation, DNA fragmentation, and membrane blebbing.

Joung et al. conducted a study utilizing several cytokines to attack A375 melanoma cells that overexpress candidate genes. Their findings in vitro revealed that both myeloid cell leukemia sequence 1 protein (MCL1) and JunB proto-oncogene (JUNB) encode proteins belonging to the Bcl-2 family, which function to inhibit apoptosis and enhance drug resistance. Notably, JUNB encodes a transcription factor that is capable of downregulating FasL and TRAIL receptors, while simultaneously upregulating BCL2-related protein A1 (BCL2A1). Additionally, JUNB interferes with the expression of genes linked to the nuclear factor-kappa B (NF-κB) complex, activates the NF-κB signaling pathway, and consequently inhibits apoptosis in melanoma cells [25]. Previous studies by Wu et al. have verified that multiple compounds can promote melanoma cell apoptosis by activating the TRAIL/death receptor 5 (DR5) apoptotic signaling pathway. Since both TRAIL and arginine deiminase pegylated 20 (ADI-PEG20) exhibit negative regulatory effects on melanoma, their combination can significantly enhance cytotoxicity against melanoma cells. Specifically, ADI-PEG20 increases the expression of pro-apoptotic protein Noxa and DR4/5 while reducing the expression of inhibitor of apoptosis protein (IAP), thereby making the cells more susceptible to TRAIL and promoting melanoma cell apoptosis [26]. Jazirehi et al. confirmed that histone deacetylase inhibitors (HDACi) not only up-regulated the expression of TRAIL and DR5 to activate the TRAIL/DR5 apoptotic signaling pathway but may also promote the recognition and killing of melanoma cells by cytotoxic T lymphocytes (CTL), further enhancing apoptosis in vitro [27]. Additionally, low-dose interferon-β (IFN-β) combined with TRAIL affected the expression of a series of apoptosis-related proteins, activated the Caspase pathway, and induced apoptosis in multiple melanoma cell lines [28]. In preclinical mouse models, the combination of Aurora kinase A inhibitor alisertib and TRAIL effectively inhibited melanoma tumor growth. Experiments in vitro showed that the combined treatment increased the recruitment of death receptor-associated signaling molecules, such as FADD in melanoma cells, activated Caspases, and triggered downstream Caspase cascade reactions, ultimately leading to apoptosis [29]. FK506-binding protein 51 (FKBP51), a pro-cancer factor associated with melanoma cells related to drug resistance and epigenetic changes, has been shown to increase the expression of TRAIL-R2 when silenced, thereby enhancing melanoma cell sensitivity to TRAIL-induced apoptosis in vitro [30]. 5-aza-2′-deoxycytidine (5-Aza) induced melanoma cell apoptosis by upregulating tumor necrosis factor (TNF)-α, Fas ligand (FasL), and TNF-related apoptosis-inducing ligand (TRAIL), demonstrating anti-melanoma effects with minimal impact on normal human melanocytes in vitro [31]. The potential mechanisms of apoptosis during melanoma progression are shown in Figure 1. Active components from traditional Chinese medicine can also exert anti-melanoma effects by promoting apoptosis. Huang et al. found that baicalein and baicalin, the major flavonoids derived from the edible medicinal plants *Scutellaria baicalensis*, induced melanoma apoptosis in vitro and in vivo by inhibiting glucose uptake and metabolism in tumor cells and affecting the target of rapamycin (TOR)-hypoxia-inducible factor-1α (HIF-1α) signaling pathway [32].

Additionally, in melanoma tissues, high expression of Bcl-2 was associated with poor prognosis in patients. This correlation may be due to elevated levels of Bcl-2 protein inhibiting the oligomerization of Bax and Bak within cells, thereby blocking the intrinsic apoptosis pathway, making tumor cells more likely to evade immune surveillance and clearance, and promoting tumor progression and metastasis [33]. Wang et al. confirmed that hernandezine, a bisbenzylisoquinoline alkaloid extracted from the traditional Chinese herbal medicine *Thalictrum glandulosissimum*, up-regulated Bax but down-regulated Bcl-2, thereby increasing the activation and cleavage of apoptosis-related proteins such as Caspase-3 and Caspase-9, ultimately inducing apoptosis in melanoma cells such as A375 and B16 in vitro [34]. Taken together, regulating the apoptotic signaling pathway may represent an effective strategy for combating melanoma.

## 3. Autophagy in Melanoma

Autophagy is a highly conserved cellular self-degradation process that plays a crucial role in maintaining intracellular homeostasis through the removal of damaged organelles and misfolded proteins. The biological features of autophagy are characterized by organelle swelling, an amorphous cytoplasmic appearance, nuclear fragmentation, pyknosis, the presence of numerous phagocytic vesicles, and membrane blebbing. Morphological changes associated with autophagy include the formation of phagophores, the closure of autophagosomes, and the fusion of autophagosomes with lysosomes [35,36]. Furthermore, autophagy exhibits a dual role in cancer biology, as it can inhibit tumor initiation while simultaneously promoting tumor progression [37].

According to the pathways by which cellular materials are transported to lysosomes, autophagy can be classified into macroautophagy, microautophagy, and chaperone-mediated autophagy. Currently, macroautophagy, the most extensively studied form, is often simply referred to as autophagy [38]. Autophagy is a complex self-degradation process: when intracellular nutrients are sufficient, the mammalian target of rapamycin complex 1 (mTORC1) phosphorylates Ser757 of UNC-51 like kinase 1 (ULK1) and autophagy-related protein 13 (ATG13), thereby inhibiting the initiation of autophagy. However, under conditions of nutrient deficiency or cellular stress, adenosine monophosphate-activated protein kinase (AMPK) becomes activated and phosphorylates Ser317 and Ser777 of ULK1 to counteract the inhibitory effects of mTORC1, thereby promoting the initiation of autophagy [39]. In detail, ULK1 activates the phosphatidylinositol 3-kinase (PI3K)-III complex formed by PI3K, Beclin 1 and VPS34, facilitating the formation of phagophores with the assistance of the ATG complex [40,41]. Ultimately, autophagosomes fuse with lysosomes to form autolysosomes, where their contents are subsequently degraded by hydrolases for recycling [42]. In melanoma, autophagic activity is regulated by oncogenic signals such as BRAF and NRAS and is closely associated with tumor metabolic reprogramming.

**Figure 2:**
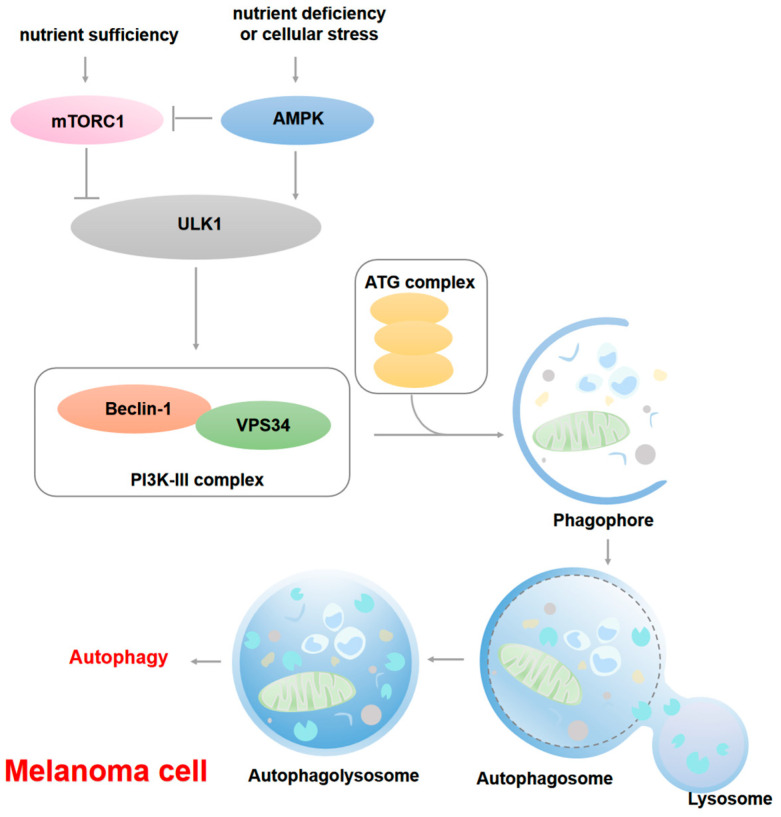
Potential molecular mechanisms of autophagy in melanoma. When intracellular nutrients are sufficient, mammalian target of rapamycin complex 1 (mTORC1) phosphorylates serine 757 of human serine/threonine-protein kinase ULK1 (ULK1) and autophagy-related protein 13 (ATG13), thereby inhibiting the initiation of autophagy. Under conditions of nutrient deficiency or cellular stress, adenosine monophosphate-activated protein kinase (AMPK) becomes activated and phosphorylates serine 317 and serine 777 of ULK1. This phosphorylation by AMPK counteracts the inhibitory effects of mTORC1, thereby promoting the initiation of autophagy. In detail, ULK1 activates the phosphatidylinositol 3-kinase (PI3K-III) complex, which includes Beclin-1 and VPS34, facilitating the formation of phagophores with the assistance of the ATG complex. Ultimately, autophagosomes fuse with lysosomes to form autolysosomes, where their contents are subsequently degraded by hydrolases for recycling.

Autophagy plays a dual role by inhibiting tumor initiation while simultaneously supporting tumor progression [37]. On one hand, it promotes tumor cell survival, enhances drug resistance, and facilitates tumor advancement; on the other hand, it induces cell death under specific conditions. During the precancerous stage, the inhibition of autophagy leads to reactive oxygen species (ROS) accumulation and genomic dysfunction, collectively increasing ER stress and promoting DNA damage, thereby suppressing tumor formation [43]. However, under conditions of starvation or oxidative stress, autophagy provides essential energy and nutrients for tumors, promoting progression, metastasis, drug resistance, and immune escape [44,45,46]. Frangež et al. have shown that compared to benign nevi, primary tumors from melanoma patients exhibit significantly reduced expression of key autophagy-related genes (ATG) including *ATG5* and *ATG7* [47]. Notably, reduced ATG5 expression has been confirmed to be closely associated with shortened progression-free survival in early-stage melanoma patients. The knockout of ATG5 or ATG7 significantly inhibited the survival of melanoma cells under low-nutrient conditions [48,49]. In addition, melanoma cells in hypoxic and nutrient-deficient microenvironments degrade organelles and macromolecules through autophagy to generate metabolic precursors such as adenosine triphosphate (ATP), amino acids, and fatty acids, maintaining tumor survival advantages under stress [50]. In a mouse model of melanoma, ATG7 gene deletion accelerated disease onset and reduced overall survival, indicating that autophagic defects caused by ATG7 deficiency may counteract oncogene-induced senescence and promote melanoma development [51,52]. In the late stages of melanoma progression, autophagy promotes tumor cell survival and contributes to drug resistance. For instance, B-Raf proto oncogene Serine/Threonine protein kinase (*BRAF*) induces ER stress and up-regulates autophagy, rendering melanoma cells resistant to chemotherapy [53]. Induced autophagy has also been observed in melanoma patients undergoing BRAFV600E-targeted therapy [54,55]. The above evidence indicates that autophagy helps melanoma cells survive during treatment, presenting a significant challenge for cancer therapy. The potential mechanisms of apoptosis during melanoma progression are shown in Figure 2.

Based on the association between autophagy and melanoma, drugs targeting autophagy have emerged as potential therapeutic strategies for melanoma, with current efforts mainly focusing on the development and application of autophagy inhibitors. DCC-3116 has demonstrated significant inhibitory effects on both ULK1 and ULK2, exhibiting notable antitumor efficacy, that has progressed to the early stages of clinical trials [56]. The most commonly used autophagy inhibitors in clinical practice are the antimalarial drugs chloroquine (CQ) and its derivative hydroxychloroquine (HCQ). These drugs exert anti-melanoma effects by alkalinizing the acidic environment of lysosomes, disrupting autolysosome formation, and inhibiting autophagic flux [57,58,59]. Sun et al. found that sinomenine inhibited the growth of melanoma by promoting autophagy via Beclin-1 enhancement in vitro and in vivo [60]. Therefore, a comprehensive consideration of their multiple mechanisms is essential when evaluating the anticancer effects of CQ/HCQ.

## 4. Pyroptosis in Melanoma

Pyroptosis represents a specific type of programmed cell death that is facilitated by the Gasdermin protein family [61]. This process is distinguished by cellular swelling, the formation of pores in the plasma membrane, and the subsequent release of pro-inflammatory cytokines, which collectively contribute to the induction of cell death in melanoma.

Gasdermin D (GSDMD), a crucial executioner molecule of pyroptosis, forms membrane pores through its N-terminal domain following cleavage, leading to osmotic cell swelling, plasma membrane rupture, and cell death [61]. Compared with apoptosis, pyroptosis occurs more rapidly and vigorously, accompanied by the release of numerous pro-inflammatory factors. The inflammasome consists of intracellular pattern recognition receptors (PRRs), apoptosis-associated speck-like protein containing a caspase recruitment domain (ASC), and inflammatory caspases (e.g., Caspase-1). The most common PRRs include nucleotide-binding oligomerization domain (NOD)-like receptors (NLRs, such as NLRP1, NLRP3, and NLRC4) and Absent in Melanoma 2 (AIM2). These intracellular PRRs can be activated by various stimuli, including bacteria, viruses, toxins, RNA, and other pathogen-associated molecular patterns (PAMPs) or damage-associated molecular patterns (DAMPs). Pyroptosis is triggered by multiple signaling pathways, primarily including the classical inflammasome pathway, non-classical inflammasome pathway, apoptotic caspase-mediated pathway, and granzyme-mediated pathway [62]. In the classical inflammasome pathway, PAMPs or DAMPs activate inflammasomes, leading to the recruitment and activation of Caspase-1. Upon the activation of Caspase-1, this enzyme is responsible for the precursors of interleukin-1β (IL-1β) and interleukin-18 (IL-18) into their biologically active forms. Additionally, it cleaves GSDMD to produce its active fragment, GSDMD-N. The GSDMD-N fragment translocates to the plasma membrane, where it oligomerizes to form membrane pores. This process results in cell swelling and eventual rupture, which facilitates the release of mature IL-1β and IL-18, as well as potassium ions, thereby further enhancing the inflammatory response [63,64]. The non-classical inflammasome pathway is often activated by lipopolysaccharides from Gram-negative bacteria, directly binding and activating Caspase-4/5/11 to cleave GSDMD and induce pyroptosis, while simultaneously activating the NLR family pyrin domain-containing 3 (NLRP3) inflammasome to promote cytokine maturation and release [65,66]. In the apoptotic caspase-mediated pathway, various chemotherapeutic agents and TNF can initiate the activation of apoptotic caspases, including Caspase-3 and Caspase-8. When cells express the relevant gasdermin proteins, such as GSDME, GSDMC, and GSDMB, these proteins undergo cleavage. This cleavage results in the formation of gasderimin pores which subsequently trigger the process of pyroptosis [62,67,68]. In the granzyme-mediated pathway, granzymes, including granzyme A (GZMA) and granzyme B (GZMB), derived from natural killer cells or cytotoxic T lymphocytes, enter target cells via perforin, cleaving specific members of gasdermin family including GSDMB and GSDME to induce pyroptosis in cancer cells, thereby enhancing the inflammatory response within the tumor microenvironment and promoting antitumor immunity [69]. *DFNB59* was the first reported gene that leads to deafness via neuronal dysfunction along the auditory cascade [70]. Aside from deafness, DFNB59 is also a core gasdermin family member responsible for pyroptosis with an N-terminal effector domain and a C-terminal inhibitory domain. Under normal conditions, the C-terminal inhibitory domain suppresses the pore-forming activity of the N-terminal domain. Upon stimulation, gasdermin proteins are cleaved by upstream-activated caspases or granzymes, releasing the N-terminal domain, which forms pores in the cell membrane, leading to the release of cellular contents, cell swelling, membrane rupture, and ultimately pyroptosis [71]. Currently, novel strategies to activate or enhance specific pyroptosis pathways offer promising perspectives for boosting cancer immunotherapy. For example, Wang et al. developed a bioorthogonal system to reveal antitumour immune function of pyroptosis [72]. Zhang et al. confirmed that tumor GSDME acts as a tumor suppressor by activating pyroptosis, enhancing anti-tumor immunity [73].

**Figure 3:**
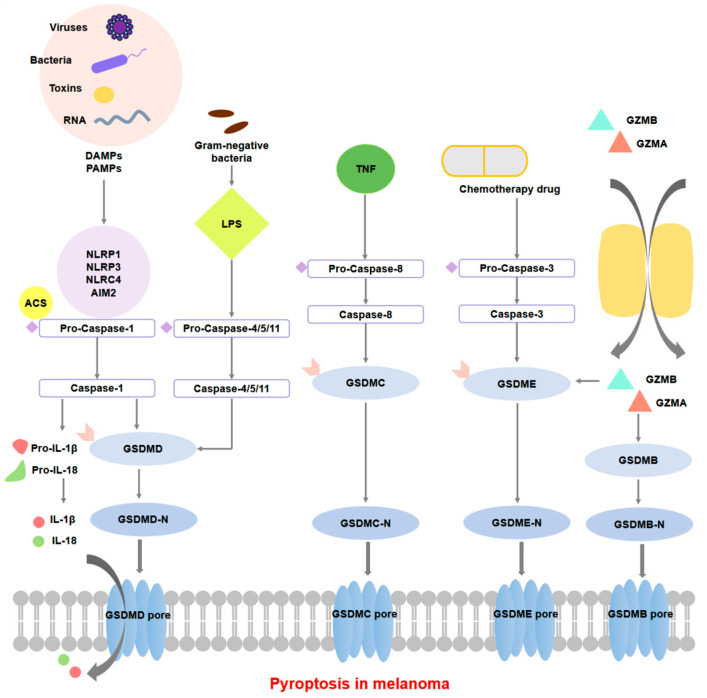
Potential molecular mechanisms of pyroptosis in melanoma. PAMPs/DAMPs are recognized by PRRs such as NLRs and AIM2, which bind to the ASC adaptor protein to recruit and activate Caspase-1. Activated Caspase-1 cleaves GSDMD, releasing its N-terminal domain (GSDMD-N), which oligomerizes to form pores in the cell membrane, leading to osmotic imbalance and membrane rupture. Concurrently, Caspase-1 processes pro-IL-1β and pro-IL-18 into their active forms, which are released through these membrane pores to trigger robust inflammatory responses. In non-canonical pathways, LPS directly activates Caspase-4/5/11, which cleave GSDMD. Under apoptotic signals, Caspase-3 cleaves GSDME while Caspase-8 cleaves GSDMC, both cleaved gasdermin proteins induce pyroptosis-like cell death. In the granzyme-mediated pathway, granzymes (e.g., GZMA and GZMB) enter target cells via perforin and cleave specific members of the gasdermin family (e.g., GSDMB, GSDME), thereby inducing pyroptosis in cancer cells. All the cleaved gasdermin proteins mentioned above can form gasdermin pores in melanoma cells.

Zaffaroni et al. have demonstrated that the activation of the classical Caspase-1/11 pathway induces the cleavage of GSDMD, releasing IL-1β and IL-18 to reshape the tumor microenvironment (TME), promote dendritic cell maturation, and enhance cytotoxic T lymphocyte infiltration [74]. BRAF inhibitors (BRAFi) and MEK inhibitors (MEKi) consitute a pharmacological combination that has received approval from the Food and Drug Administration (FDA) for the treatment of patients diagnosed with BRAF-mutated melanoma. The antitumor effectiveness of this combination facilitates the activation of Caspase-3 and GSDME, thereby inducing pyroptosis [75]. Cai et al. utilized specific siRNA to reduce the levels of recombinant phosphoinositide-dependent protein kinase 1 (PDPK1) in melanoma cells, which increased the sensitivity of these cells to antitumor drugs [76]. In BRAF inhibitor-resistant melanoma cells, small molecules such as the Caspase-3 agonist raptinal reversed the resistant phenotype by restoring GSDME expression, significantly enhancing tumor cell sensitivity to chemotherapeutic agents [77]. The emerging nanodelivery system, nanoscale CRISPR scaffold (Nano-CD), can release cisplatin and CRISPR/dCas9 plasmids on demand in the acidic intracellular environment, triggering pyroptosis in tumor cells. Additionally, Nano-CD combined with PD-1 inhibition suppresses recurrence and lung metastasis of malignant melanoma, exhibiting a robust systemic antitumor immune response and a durable immune memory effect [78]. The potential mechanisms of pyroptosis during melanoma progression are shown in Figure 3.

## 5. Immunogenic Cell Death in Melanoma

Immunogenic cell death (ICD) represents a distinct form of RCD that has the capacity to elicit adaptive immune responses in immunocompetent syngeneic hosts [79]. Rather than merely resulting in cellular demise, ICD initiates adaptive immune responses through interactions at two levels: the dying cells and the host immune system. At the cellular level, ICD is characterized by the presence of antigens on the dying cells that are recognizable by the host, as well as the release or exposure of immune-stimulating signals during the process of cell death. At the level of the host immune system, it is essential that the host maintains an intact central tolerance mechanism, which implies that clonal deletion of antigens from the dying cells has not occurred. Furthermore, the host must possess naive T-cell clones that are capable of recognizing the antigens presented by the dying cells, which are subsequently activated by the adjuvant effects of DAMPs [80,81]. ICD is probably the most studied and increasingly important mechanism involved in melanoma.

ICD can activate the host’s adaptive immune response to novel antigenic epitopes and DAMPs generated by tumor cells or virus-infected cells. This process recruits dendritic cells (DCs), activates T cells, and promotes enduring anti-tumor immunity [13,82,83]. ICD can be triggered by various stressors, including pathogens, chemotherapeutic agents, targeted anticancer compounds, and physical modalities such as photodynamic therapy. Key DAMPs involved in ICD include ATP, annexin A1 (ANXA1), endoplasmic reticulum chaperones such as calreticulin (CALR) and heat shock proteins (HSPs), high-mobility group box 1 (HMGB1), and IFNs, all of which help recruit and activate DCs. In detail, ATP functions as a “find me” signal for immune cells, while ANXA1 guides DCs to cancer cells. CALR and HSPs act as ‘eat-me’ signals for DCs, facilitating the uptake of cellular debris by DCs through low-density lipoprotein receptor related protein 1 (LRP1, also known as CD91). Meanwhile, HMGB1 enhances inflammation and antigen presentation via Toll-like receptor 4 (TLR4). Additionally, IFNs are secreted in response to RNA or DNA species, further boosting immune responses [81,84,85,86]. Collectively, these signals promote both adaptive and innate immune responses, contributing to an effective anti-tumor immune response.

IMMUNEPOTENT CRP (ICRP), a mixture of substances of low molecular weight obtained from bovine spleens, enhances the efficacy of the antitumor drug oxaliplatin in promoting ICD and inhibiting the growth of melanoma [87]. Glucose-6-phosphate dehydrogenase (G6PD) serves as the rate-limiting enzyme in the pentose phosphate pathway (PPP), playing a crucial role in the production of NADPH, which is essential for maintaining cellular redox balance. Research conducted by Nakamura et al. demonstrated that the inhibition of G6PD, either through the chemical inhibitor 6-AN or via gene knockdown, resulted in the induction of cell death in melanoma cells in vitro. Furthermore, when combined with an anti-PD-L1 antibody in vivo, this approach significantly decreased melanoma tumor volume. The underlying mechanism is attributed to G6PD inhibition, which induces ICD and facilitates the release of tumor antigens while simultaneously enhancing T-cell activation and the antigen-presenting capabilities of DCs, thereby addressing resistance to immune checkpoint inhibitors [88]. In another study, Le et al. developed manganese zinc sulfide nanoparticles that promote immunogenic cell death, reprogram the tumor microenvironment (TME), and stimulate anti-tumor immune responses, thereby improving treatment outcomes for metastatic melanoma [89]. According to human leukocyte antigen (HLA), Ott et al. introduced an immunogenic personal neoantigen vaccine for patients with melanoma. This vaccine effectively circumvents tumor immune evasion mechanisms, activates targeted immune responses against tumor cells, induces ICD, and enhances therapeutic efficacy [90]. Additionally, Tartrolon D (TRL), a symbiotic cellulose-degrading bacterium, has been shown to inhibit melanoma cells in a dose-dependent manner. Mechanistic investigations revealed that TRL-treated B16-F10 melanoma cells exhibited a significant increase in the expression of major histocompatibility complex (MHC) class II molecules and CD1d, thereby improving the efficiency of tumor antigen presentation. In a C57BL/6 mouse model, TRL-induced ICD was found to promote splenocyte activation, indicating its potential to elicit a systemic anti-tumor immune response [91]. The in vivo and in vitro study conducted by Rossi et al. demonstrated that nanosecond pulsed electric fields (nsPEF) efficiently promoted ICD in B16F10 melanoma tumors [92]. The simultaneous reestablishment of the p53/p19Arf and interferon-β signaling pathways in melanoma cells leads to ICD, triggering an anti-tumor immune response involving natural killer cells, neutrophils, and both CD4^+^ and CD8^+^ T lymphocytes [93]. Zhou et al. demonstrated that the combination of carbon ion radiotherapy and anti-PD-1 therapy significantly reduced tumor growth and extended the survival of mice with melanoma. This effect was attributed to the induction of ICD, which enhanced the immunogenicity of the tumor and improved the effectiveness of subsequent anti-PD-1 immunotherapy [94].

Furthermore, Ren et al. established that utilizing an ICD-dependent risk signature (ICDRS) to predict responses to immunotherapy and targeted drug therapies could be beneficial for various risk subpopulations of patients diagnosed with melanoma [95]. The aforementioned studies suggest that the induction of ICD presents a significant opportunity to augment the immunogenicity of tumor cells, thereby facilitating an enhanced immune amplification response, increasing sensitivity to therapeutic agents, and ultimately contributing to melanoma treatment.

## 6. Necroptosis in Melanoma

**Figure 4:**
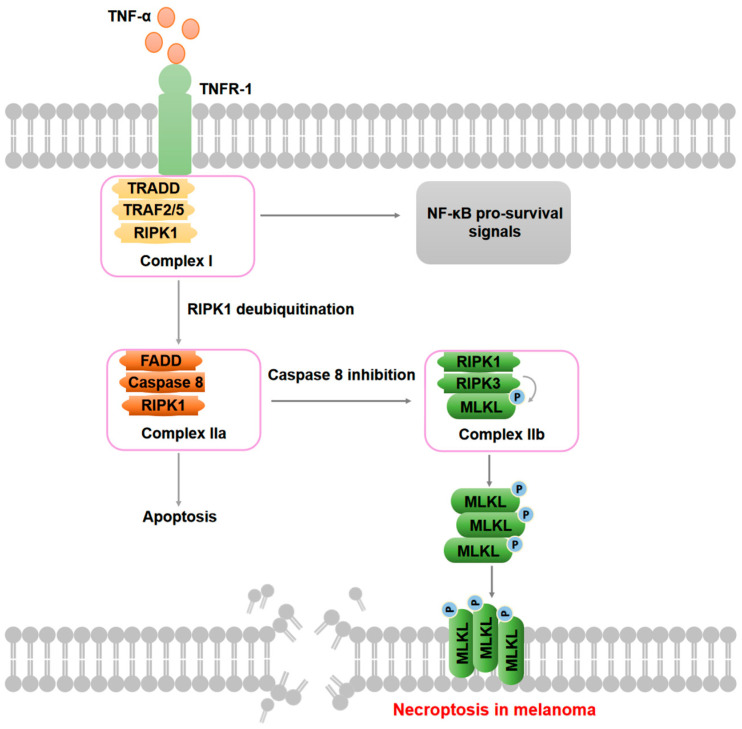
Potential molecular mechanisms of necroptosis in melanoma. Upon binding to TNF-α, TNFR1 recruits TNF receptor type 1-associated death domain protein (TRADD), TRAF2/5, and RIPK1 to form complex I, activating NF-κB pro-survival signals. However, when RIPK1 is deubiquited, it dissociates from complex I and forms complex IIa with FADD and caspase-8, which will then cause apoptosis. If caspase-8 is inhibited, RIPK1 activates RIPK3, which subsequently phosphorylates MLKL to form complex IIb, followed by MLKL oligomerization and translocation to the plasma membrane, resulting in ion imbalance, membrane integrity disruption, and ultimately cell swelling and rupture.

Necroptosis represents a distinct form of programmed necrotic cell death independent of caspases, while exhibiting morphological features of necrotic cells and signaling mechanisms similar to those of apoptosis. Morphologically, necroptosis is characterized by several features, including perforation of the cell membrane, increased intracellular osmotic pressure, cell rounding and swelling, organelle swelling, mitochondrial dysfunction, loss of mitochondrial membrane potential, alterations in nuclear chromatin, progressive membrane rupture, and the release of DAMPs, mitochondrial DNA, cellular contents, and membrane lipid, which can exacerbate peripheral inflammatory responses. Notably, apoptotic bodies are absent in necroptosis [96,97]. In comparison to ICD, necroptosis is characterized by the release of a greater number of substances; however, it is deficient in specific signals that activate the immune response. Unlike necrosis, necroptosis is governed by strict intracellular signal regulation and actively utilizes ATP. The principal signaling pathway involved in necroptosis comprises receptor-interacting protein kinase 1 (RIPK1), RIPK3, and mixed lineage kinase domain-like protein (MLKL), with its activation depending on signals from death receptors (e.g., TNFR1), Toll-like receptors, or intracellular DNA sensors (e.g., ZBP1) [98,99,100,101]. Taking TNF-α-induced necroptosis as an example, upon binding to TNF-α, TNFR1 recruits TNF receptor type 1-associated death domain protein (TRADD), TRAF2/5, and RIPK1 to form complex I, which activates NF-κB pro-survival signals. However, when RIPK1 is deubiquited, it dissociates from complex I and assembles complex IIa with FADD and caspase-8. If caspase-8 is inhibited, RIPK1 subsequently recruits and activates RIPK3, leading to the formation of a necroptosome (complex IIb) [102]. The activated RIPK3 then phosphorylates Thr357/Ser358 of MLKL, which triggers MLKL oligomerization and translocation to the cell membrane, disrupts ion balance, compromises membrane integrity, and ultimately results in cell swelling and rupture [103,104]. Furthermore, Yan et al. revealed a critical role of necroptosis in tumorigenesis and metastasis, suggesting the potential for targeting necroptosis as a novel therapeutic strategy in cancer treatment [105]. The potential mechanisms of necroptosis during melanoma progression are shown in Figure 4.

Current studies have revealed abnormalities in the regulatory mechanisms of necroptosis in melanoma. Utilizing single-cell sequencing analysis and bulk-RNA sequencing analysis highlighted the complex role of necroptosis in cutaneous melanoma [106]. Evidence indicates that RIPK3 is expressed at lower levels in melanoma cell lines, but higher in normal melanocytes and benign nevi [107]. This suggests that melanoma cells may evade necroptosis by reducing the expression of these regulators. Recently, Bak et al. developed a type of human adipose-derived stem cells (ADSCs) that incorporates the RIPK3 gene, referred to as RP@ADSCs. RP@ADSCs-mediated immunotherapy was showed in mouse models harboring K1735 melanoma cells, suggesting there potential as a viable therapeutic agent for anti-melanoma treatment in clinical settings [108]. Additionally, a novel RIPK1 inhibitor PK68 significantly suppresses lung metastasis of mouse melanoma cells [109]. However, it is important to note that in addition to mediating necroptosis, RIPK1 can also participate in regulating endoplasmic reticulum stress and autophagy [110]. This indicates that the role of RIPK1 in melanoma cells is complex. Researchers have found that transforming growth factor-β-activated kinase 1 (TAK1) can inhibit RIPK1 activity. Therefore, even when RIPK3 and MLKL are normally expressed, TAK1 can still prevent melanoma cell death [111]. The above studies imply that RIPK1 may act as an upstream mediator of necroptosis in melanoma cells, suggesting that targeting necroptosis for anti-melanoma therapy requires further in-depth exploration focusing on RIPK1.

Despite the mechanisms by which melanoma cells evade necroptosis, specific strategies can reactivate this process to exert antitumor effects. Both in vivo and in vitro studies have shown that combining pan-caspase inhibitors with radiotherapy, dacarbazine, and hyperthermia increased the infiltration of dendritic cells and CD8^+^ T cells in the tumor microenvironment to induce necroptosis and inhibit melanoma growth [112]. Prior investigations in vivo by Van Hoecke et al. demonstrated that the intratumoral administration of mRNA encoding MLKL effectively impeded the growth and metastasis of melanoma in mouse models, displaying a synergistic interaction with anti-PD-1 immunotherapy [113]. Conversely, an alternative in vivo study by Martens et al. confirmed that MLKL deficiency resulted in a reduced growth rate of nevi and diminished infiltration and expansion of melanoma cells within the inguinal lymph nodes of male mice. However, no significant differences were observed in the melanoma development rate among female mice [114]. The discrepancies in these findings may be attributed to variations in genetic backgrounds, sex, and methodologies employed for tumor induction.

## 7. Ferroptosis in Melanoma

Ferroptosis represents a form of programmed cell death that is initiated by iron-dependent lipid peroxidation, involving intricate mechanisms that intersect with iron metabolism, lipid peroxidation, and glutathione metabolism, ultimately leading to the death of melanoma cells [115].

Ferroptosis is primarily characterized by mitochondrial shrinkage, a reduction in cristae, and an increase in membrane density, all of which are accompanied by lipid peroxidation and subsequent rupture of the cell membrane. In the context of iron metabolism, cellular uptake of iron occurs via transferrin receptors (TFR), leading to its accumulation, which subsequently triggers Fenton reactions and results in the generation of ROS [116]. Lipid peroxidation, the central event in ferroptosis, predominantly involves the oxidation of membrane constituents, such as polyunsaturated fatty acids and phosphatidylethanolamine, culminating in the formation of cytotoxic lipid peroxides [117]. The metabolism of glutathione (GSH) is critical for sustaining cellular antioxidant defenses. A reduction in GSH levels weakens the activity glutathione of peroxidase 4 (GPX4), thereby impairing the clearance of lipid peroxides and facilitating ferroptosis [118]. The GPX4-dependent antioxidant pathway, particularly the solute carrier family 7 member 11 (SLC7A11)/GSH/GPX4 axis, functions as an intracellular protective mechanism, relying on GPX4 to mitigate excessive iron-dependent free radical production, inhibit lipid peroxidation, and safeguard cell membranes from oxidative damage—an essential process for maintaining redox homeostasis and preventing ferroptosis [119]. Furthermore, GPX4-independent antioxidant systems also contribute to the regulation of ferroptosis. Additionally, nuclear factor erythroid 2-related factor 2 (NRF2), a pivotal transcription factor involved in cellular responses to oxidative stress, protects cells from ROS-induced damage by promoting the expression of downstream target genes, including *GPX4*, ferritin heavy chain 1 (*FTH1*), ferroptosis suppressor protein 1 (*FSP1*), glutamate-cysteine ligase (*GCL*), and glutathione synthetase (*GS*) [120]. Consequently, the induction of ferroptosis has the potential to inhibit tumor growth, thereby contributing to the suppression of tumorigenesis.

In melanoma, multiple ferroptosis-related proteins play critical roles. Cytoglobin (Cygb), an endogenous antioxidant protein, exhibits a dual role in melanoma. Zou et al. demonstrated that Cygb inhibited melanoma invasion and metastasis by scavenging ROS and regulating lipid metabolism, while also protecting tumor cells from ferroptosis by maintaining glutathione levels [121]. Arginase 2 (Arg2) promoted GPX4 expression and inhibited lipid peroxidation, whereas sorafenib downregulated Arg2 expression to induce ferroptosis in melanoma [122]. In circulating tumor cells (CTCs) from melanoma patients, the lipogenesis regulator SREBP2 directly induced transferrin transcription and inhibited ferroptosis by reducing intracellular free iron, ROS, and lipid peroxidation levels to enhance the survival and drug resistance of CTCs [123]. All of the aforementioned critical proteins involved in ferroptosis have been regarded as the candidate targets for melanoma.

As a classical multi-tyrosine kinase inhibitor, sorafenib promotes ferroptosis in melanoma cell by inhibiting SLC7A11 activity and depleting glutathione (GSH) [124]. In vemurafenib-resistant non BRAF-mutated melanoma cells, sorafenib increased intracellular ROS levels and induced ferroptosis, thereby enhancing the sensitivity of resistant tumors to vemurafenib. The combined use of these two agents significantly inhibited the viability of vemurafenib-resistant melanoma cells, which was impeded by the ferroptosis inhibitor ferrostatin-1 [125]. The ferroptosis inducer erastin exerts cytotoxic effects on melanoma cells via ferroptotic mechanisms without significantly affecting immune activity. When used in conjuction with oncolytic viruses, erastin synergistically enhances antitumor immune responses and therapeutic efficacy by promoting the proliferation of activated dendritic cells and enhancing the activity of tumor-specific CD8^+^ T cells [126]. Salicylazosulfapyridine (SSZ), a common anti-inflammatory drug, induces ferroptosis in melanoma cells by upregulating the expression of prostaglandin endoperoxide synthase 2 (PTGS2) and acyl-CoA synthase long-chain family member 4 (ACSL4), inhibiting the synthesis of GPX4 and FTH1, and promoting intracellular lipid peroxidation [126]. Sulfasalazine, which functions as an inhibitor of the cystine–glutamate antiporter, has been shown to reduce intratumoral levels of glutathione. This reduction subsequently increases the sensitivity of B16F10 melanoma cells to radiation therapy [127]. Taken together, ferroptosis-inducing agents (FINs) exhibit unique therapeutic potential in the treatment of melanoma.

**Figure 5:**
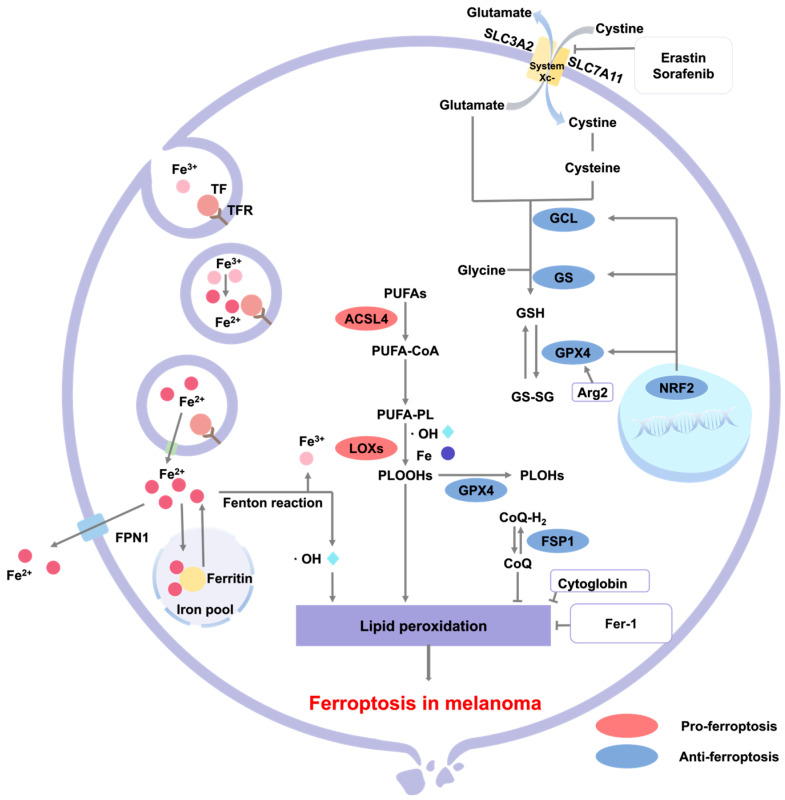
Potential molecular mechanisms of ferroptosis in melanoma. Ferroptosis is regulated by three primary pathways: iron metabolism, the classical GPX4-regulated pathway, and lipid peroxidation metabolism. Ferric/ferrous ions (Fe^3+^/Fe^2+^) enter cells via TFR-mediated endocytosis, generating ·OH through the Fenton reaction to trigger lipid peroxidation. The system Xc⁻-mediated cystine uptake and GSH synthesis inhibit lipid peroxidation, while GPX4 maintains cellular redox balance by reducing phospholipid hydroperoxides (PLOOHs). Pro-ferroptotic factors (e.g., ACSL4, lipoxygenases [LOXs]) and anti-ferroptotic factors (e.g., GPX4, NRF2, FSP1) collectively regulate the ferroptotic process in melanoma. The figure also indicates relevant drug targets and inhibitors (e.g., Erastin and Fer-1).

The emergence of nanomedicines has opened a new pathway for the treatment of melanoma. Compared to traditional FINs, nanomedicines targeting ferroptosis offer numerous advantages. They can achieve both active and passive targeting of tumors, significantly improve the solubility and bioavailablility of drugs, and enable the sustained drug release. For instance, Xie et al. have developed a phototheranostic metal–phenolic network, encapsulating the ferroptosis inducer Fe^3+^ and the exosome inhibitor GW4869 within a semiconductor polymer. This compound not only exerts photothermal therapy to induce tumor necrosis and ICD but also releases Fe^3+^ to subsequently trigger tumor ferroptosis. Concurrently, the released GW4869 inhibits exosomal programmed death-ligand 1 (PD-L1), promotes lipid peroxidation and T cell activation, and suppresses the growth of melanoma [128]. Another study by Wang et al. has prepared a hepatocyte growth factor (HGF) nanounit, encapsulating GW4869 and Fe^3+^ with a hyaluronic acid. This nanounit can effectively reduce the secretion of PD-L1 by melanoma cells, relieve the inhibition of T cell activity, and enable T cells to enhance the secretion of immunologically active IFN-γ. The Fe^3+^ component in the HGF nanounit can induce lipid peroxidation and promote ferroptosis. In in vivo experiments, the HGF nanounit not only inhibits tumor growth but also stimulates the generation of cytotoxic T cells and immune memory. When combined with anti-PD-L1 antibodies, it can overcome the limitations associated with free antibody treatments and effectively inhibit tumor metastasis [129]. Taken together, the potential molecular mechanisms of ferroptosis in melanoma are shown in Figure 5.

## 8. Limitations and Future Perspectives

There are relatively few reports on RCD across different classifications of melanoma. Therefore, further research on the exact role of RCD in different types of melanomas will be helpful for exploring more specific therapeutic approaches for melanoma. In addition, at present, research efforts aimed at managing drug resistance, off-target effects, tumor heterogeneity and the immune microenvironment through the modulation of RCD-related protein expression—particularly core mediators and upstream regulatory molecules—are still in the nascent phases. There is a significant gap in effective strategies to mitigate the substantial harm inflicted on healthy cells due to the non-specific targeting of RCD in the elimination of melanoma cells.

There have been several agents targeting RCD to suppress melanoma (some of them are listed in Table 1). However, a given regulatory cell death pathway may affect the other pathways. For instance, there exists a bidirectional regulatory interaction between apoptosis and autophagy. Apoptosis-related proteins from the Bcl-2 family can inhibit autophagic activity by directly binding to Beclin-1. Additionally, apoptotic executioner Caspases can terminate the pro-survival functions of autophagy by cleaving ATG5. Conversely, autophagy can inhibit the apoptotic cascade by eliminating damaged mitochondria, thereby reducing the release of Cyto-C. However, the accumulation of ROS due to autophagic defects may paradoxically induce apoptosis through the activation of the DNA damage-p53 pathway. Necroptosis also interacts with other types of RCD. When apoptosis is inhibited and Caspase-8 is inactivated, the RIPK1/RIPK3/MLKL pathway is activated, leading to necroptosis. Autophagy can mitigate necroptosis by clearing necrosomes. Furthermore, DAMPs released during necroptosis can activate the NLRP3 inflammasome, creating a synergistic effect with pyroptosis that promotes the release of IL-1β. During pyroptosis, membrane pores formed by GSDMD facilitate lipid peroxidation, a characteristic of ferroptosis, by disrupting cellular osmotic pressure. Autophagy counteracts pyroptosis by eliminating mitochondrial ROS to inhibit NLRP3 inflammasome activation. In the context of ferroptosis, autophagy can directly promote this process by releasing free iron through ferritinophagy, and the regulation of ACSL4 by the RIPK1/TAK1 pathway indicates shared signaling nodes with necroptosis. These interactions underscore the intricate molecular dialogs and crosstalk among various forms of RCD, presenting significant challenges for the targeted regulation of RCD in the treatment of melanoma.

In addition, cuproptosis, a novel type of copper-dependent cell death, has recently been described and is associated with melanoma [130,131]. PANoptosis is also a newly discovered form of programmed cell death. It integrates the characteristics of pyroptosis, apoptosis, and necroptosis, and is a highly coordinated and dynamically balanced pathway of programmed inflammatory cell death. Utilizing bulk and single-cell transcriptome analyses, machine learning modeling, and immune correlation assessments confirmed the involvement of PANoptosis in melanoma [132]. In addition to the aforementioned types of RCD, netotic cell death, entotic cell death, lysosome-dependent cell death, parthanatos, oxeiptosis, disulfidptosis, and alkaliptosis constitute a cell death index to predict the prognosis and drug sensitivity of melanoma [133]. The targeting of PANoptosis has garnered significant interest due to its potential to influence various forms of RCD. Additionally, combination therapies that simultaneously target multiple RCD pathways for the treatment of melanoma have commenced clinical trials. Notable examples include NCT01740297, which focuses on apoptosis, pyroptosis, and necroptosis, and NCT02967692, which targets both apoptosis and pyroptosis [134,135]. Nevertheless, the specific roles of various types of RCD in melanoma are not yet fully elucidated, presenting significant obstacles to the development of targeted RCD-based therapeutic strategies for melanoma.

In terms of therapeutic strategies, combination therapy regimens based on mechanisms of cell death are likely to become a research hotspot in the future. The rational combination of traditional treatment methods and novel therapeutic approaches, such as small-molecule targeted therapy and immunotherapy, will maximize the benefits of different treatment modalities to synergistically induce melanoma cell death, enhance therapeutic efficacy, and minimize the development of drug resistance.

## Figures and Tables

**Table 1:** RCD type and key effectors of agents for melanoma.

Agents	RCD Type	Key Effectors	References
5-Aza	apoptosis	TNF-α, FasL, TRAIL	[31]
hernandezine	apoptosis	Bax, Bcl-2, Caspase-3, Caspase-9	[34]
DCC-3116	autophagy	ULK-1, ULK-2	[56]
sinomenine	autophagy	Beclin-1	[60]
BRAFi, MEKi	pyroptosis	Caspase-3, GSDME	[75]
raptinal	pyroptosis	Caspase-3, GSDME	[77]
6-AN	ICD	G6PD	[88]
tartrolon D	ICD	MHC, CD1d	[91]
RP@ADSCs	necroptosis	RIPK3	[108]
PK68	necroptosis	RIPK1	[109]
sorafenib	ferroptosis	SLC7A11	[124]
salicylazosulfapyridine	ferroptosis	PTGS2, ACSL4,GPX4	[126]

## Data Availability

No new data were created or analyzed in this study.
